# High prevalence of reflux esophagitis among upper endoscopies in Chinese patients with chronic liver diseases

**DOI:** 10.1186/1471-230X-10-54

**Published:** 2010-06-04

**Authors:** Bing Li, Bin Zhang, Jun Wei Ma, Peng Li, Lei Li, Yun Ming Song, Hui Guo Ding

**Affiliations:** 1 Department of Hepatology and Gastroenterology, Beijing You'an Hospital Affiliated with Capital Medical University, Beijing 100069, China

## Abstract

**Background:**

Reflux esophagitis (RE) is increasing in prevalence in China. There are very few studies on the prevalence and factors related to RE in patients with chronic liver diseases. The aims of this study were to determine the prevalence of RE by endoscopy in patients with chronic liver diseases and the possible related predictors of RE.

**Methods:**

A total of 1,280 patients with chronic liver disease and 29 patients with acute hepatitis A or E were prospectively evaluated. There were 879 and 401 patients with liver cirrhosis or chronic hepatitis, respectively. RE was classified by endoscopy according to the Los Angeles classification scheme.

**Results:**

RE was diagnosed in 36.4% (469/1280) of the chronic liver disease patients, which was significantly higher than in the acute hepatitis patients (10.3% [3/29], P < 0.001). RE accounted for 43.0%, 9.7%, and 60.2% of patients with liver cirrhosis, chronic hepatitis(mild and medium), and liver failure, respectively. A high prevalence of RE existed in patients with liver failure and/or Child B and C liver cirrhosis, with typical symptoms of RE in 21.3% of the patients (100/469). There was a significant relationship between gender, age, ascites, and RE.

**Conclusions:**

The high prevalence of RE among upper endoscopies of patients with severe chronic liver disease was demonstrated. Asymptomatic RE was more common in cirrhotic and liver failure patients. The role of RE in variceal bleeding, however, needs to be demonstrated.

## Background

Gastroesophageal reflux disease (GERD), a highly prevalent disorder, is defined as reflux of gastroduodenal content to the esophageus, and includes reflux esophagitis (RE) and Barrett's esophagus [1,2]. However, only one-half of GERD patients present with esophageal erosions, namely RE. The symptoms of GERD typically include dyspepsia, pyrosis, or tissue damage outside the esophagus, such as the pharynx, larynx, and trachea [3,4]. GERD is very common in Western countries; 7%~10% of adults have pyrosis daily and 30% ~ 40% have pyrosis on a monthly basis [2]. An epidemiologic survey in China showed the incidence of pyrosis and dyspepsia to be 8.97%[1]. The incidence of GERD, confirmed using 24-hour esophageal pH monitoring, is 5.77%, and RE confirmed by endoscopy is 1.92%, which is lower than in Western countries [1]. The causes and mechanisms of GERD have not been elucidated [5,6]. Patients with chronic liver disease patients, especially patients with portal hypertension and liver cirrhosis, have clinical manifestations, such as esophageal varices, ascites, and edema. Some studies have been conducted regarding the role of esophageal varices in the development of esophageal motor disorders and abnormal gastroesophageal reflux in these patients [7,8]. Ascites could be a factor promoting gastroesophageal reflux and it has been questioned whether or not reflux would favor the rupture of varices [9,10]. However, there are few studies on the prevalence of RE and factors related to RE in patients with chronic liver disease. Therefore, the aims of this study were to evaluate the prevalence of RE confirmed by endoscopy in patients with chronic liver disease patients and the factors related to RE.

## Methods

### Patients

Between January 2008 and January 2009, patients with chronic liver disease who submitted to endoscopy were prospectively evaluated. All patients came from the inpatients clinic department of Beijing Youan Hosital of Capital Medical University. We excluded patients with systemic disease related to esophageal motor disorders and/or gastroesophageal RE (progressive systemic sclerosis, diabetes mellitus, and neuromuscular disorders), alcohol abusers until 6 months before this study, and chronic users of drugs that influence esophageal motility (calcium channel blockers, theophyline, and nitrates). All patients were evaluated by the same associate chief physician according to a protocol for classifying etiology, Child-Pugh score, ascites, and GERD symptoms, such as pyrosis and/or dyspepsia. A diagnostic work-up of chronic liver disease was performed, including a clinical history, physical examination, laboratory tests, and/or liver pathology assessment according to the criteria suggested by the Chinese Medical Association for Liver Diseases in 2000 [11]. In brief, the diagnostic criteria of liver failure was based on the history of chronic liver disease, significant digestive symptoms and/or extreme fatigue, hepatic encephalopathy, and/or ascites, prothrombin activity (PTA) < 40%, and serum total bilirubin (TBIL) > 10 times the upper limit of normal. Of the enrolled patients, 29 patients with acute hepatitis A or E, who had undergone endoscopy, were considered as controls. The complications and reflux disease questionnaire (RDQ; AstraZeneca R&D, Wuxi, China), such as pyrosis and/or dyspepsia, were noted. The study was performed in accordance with the principles of the Declaration of Helsinki. The study program was explained to the patients and/or their relatives and informed consent forms were obtained. The study was approved by the Ethical Committee at Beijing You'an Hospital of Capital Medical University.

### Upper gastrointestinal endoscopy

All patients underwent upper gastrointestinal endoscopy (OlympusXQ260; Olympus, Japan) to evaluate esophageal varices (EV), classified as small (diameter, < 3 mm), medium (diameter, 3-6 mm), or large (diameter, > 6 mm)[12]. RE, if present, was classified according to Los Angeles classification standards [13], as follows: grade A, isolated erosions, the damage was limited to the folds of mucosa and the length is < 0.5 cm; grade B, confluent but not circumferential erosions, the damage is limited to the folds of mucosa, one of which is > 0.5 cm in length; grade C, confluent and circumferential erosions, the mutual integration of mucosal damage, but < 75% of the esophagus; and grade D, confluent and circumferential erosions and encroachment is > 75% of the esophageal circumference. Hiatal hernia was considered to be present if the gastroesophageal junction was 2 cm above the diaphragmatic impression.

### Data collection

All patients had blood drawn for a complete blood count and analysis of prothrombin, albumin, alanine transaminase (ALT), aspartate transaminase (AST), alkaline phosphatase, gamma glutamyl transferase, bilirubin, cholesterol, creatinine, urea nitrogen, Na^+^, Cl^-^, and K^+ ^using an OLYMPUS automatic biochemical analyzer (OLYMPUS-AU640). In addition, blood was obtained for detection of markers for hepatitis A and B (Abbott Laboratories, Abbott Park, IL, USA), and C and E (reagents from Beijing Wantai Pharmaceutical Company, Beijing, China) using ELISA. The quality control for blood tests was administered by the National Center for Clinical Laboratory.

### Data analysis

RE related to the following variables was analyzed: presence of ascites, Child-Pugh score, gender, age, and EV size. The statistical analyses were performed using SPSS software, version 13.0 for Windows. Dichotomous variable data were analyzed by the Fisher exact test and χ2 analysis. The differences were considered statistically significant when p < 0.05.

## Results

### Characteristics of patients

A total of 1,280 patients met the inclusion criteria. There were 897 males (70.1%) and 383 females (29.9%), with a mean age of 54.3 ± 10.2 years (range, 17-73 years). The diagnosis of liver cirrhosis was made by histopathologic or clinical criteria in 879 patients (66.7%). The etiology of liver cirrhosis included hepatitis B in 757 patients (86.1%), alcohol in 67 patients (7.6%), hepatitis C in 51 patients (5.8%), and cryptogenic in 4 patients (0.45%). The patients with liver cirrhosis were classified as follows: Child A, 166 patients (18.9%); Child B, 324 patients (36.9%); and Child C, 389 patients (44.3%). Ascites was present in 672 patients (76.5%) with liver cirrhosis. EV were present in 796 patients (90.5%) with liver cirrhosis. The diagnosis of chronic hepatitis was made by histopathologic or clinical criteria in 401 patients. The etiology of chronic hepatitis included hepatitis B in 334 patients (83.3%), autoimmune hepatitis in 13 patients (3.2%), hepatitis C in 21 patients (5.2%), and cryptogenic hepatitis in 33 patients (8.2%). Ninety-seven (24.2%), 201 (50.1%), and 103 patients (25.7%) had mild, moderate, and liver failure, respectively.

### Prevalence of RE

**Figure 1:**
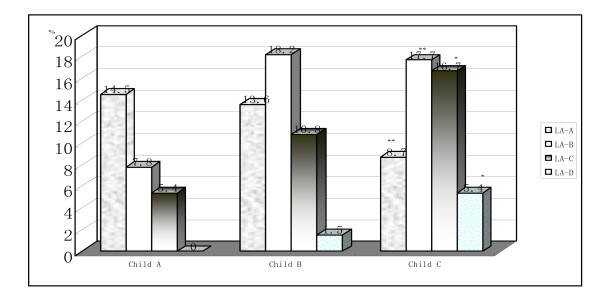
**The grade of RE in different stage cirrhotic patients**. The grade C and D of RE in Child C patients was more common than Child A or B patients, * P < 0.05~0.001. There was no difference in grade A and B of RE in cirrhotic patients with Child A, B, and C.**P > 0.05.

The prevalence of RE was 36.4% (469/1280) in patients with chronic liver disease, which was significantly higher than the control patients (10.3% [3/29], P < 0.001). RE accounted for 43.0%, 9.7%, and 60.2% of patients with liver cirrhosis, chronic hepatitis(mild and medium), and liver failure, respectively. The high prevalence of RE in patients with liver failure or Child B and C liver cirrhosis is shown in Table 1. Grades C and D of RE in Child C or liver failure patients was more common than Child A or B patients (Figure 1). Thus, there was a positive relationship between the severity of liver function damage and RE.

**Table 1:** The prevalence of RE in chronic liver disease patients

GERD^#^	Liver cirrhosis (n = 879)			Chronis hepatitis (n = 401)		
	**Child A**	**Child B**	**Child C**	**Mild**	**Medium**	**Liver failure**
	**n = 166**	**n = 324**	**n = 389**	**n = 97**	**n = 201**	**n = 103**

A	24	44	34	5	19	11
B	13	59	69	1	3	15*
C	9	35*	65**	0	1	17**
D	0	5**	21**	0	0	19**

Total	46(27.7%)	143(44.1%)	189(48.6%)*	6(6.2%)	23(11.4%)	62(60.2%)**

Compared with Child A, mild, or moderate hepatitis: *χ2 = 5.76~9.38, P < 0.05,

**χ2 = 10.6~20.33, P < 0.001. #The grade of A, B, C, and D of RE was classified by endoscopy according to Los Angeles classification standards.

### Clinical symptoms

Typical symptoms of RE were present in 21.3% of patients (100/469) and mainly occurred in patients with chronic hepatitis (43%). However, the patients with the most severe cirrhosis had asymptomatic manifestations from RE, but suffered from other symptoms. Dyspepsia and pyrosis only accounted for 12.3% and 10.4% of cirrhotic patients, respectively.

### Related variables analysis

#### Age and gender

**Figure 2:**
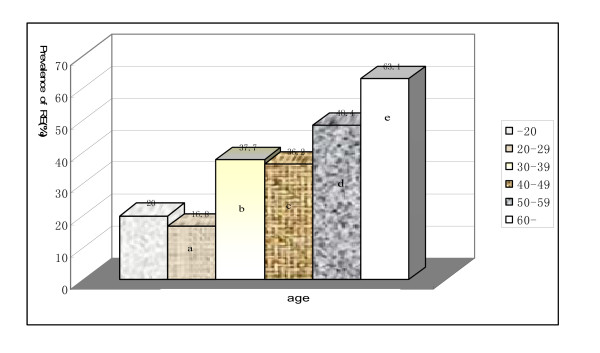
**The relationship between RE and age**. The prevalence of RE increased with age. compared with patients < 20 years of age, a: χ2 = 0.89, P = 0.66; b: χ2 = 3.65, P = 0.057; c: χ2 = 3.26, P = 0.07; d: χ2 = 8.98, P = 0.003; e: χ2 = 17.6, P = 0.0000.

RE was more common in males (43.1% [387/897]) than females (21.4% [82/383]; χ2 = 29.1, P < 0.0001). The prevalence of RE increased with age. The occurrence of RE accounted for 63.1% in patients > 60 years of age with chronic liver disease. The prevalence of RE was only 20.0% in patients < 20 years of age with chronic liver disease (Figure 2).

#### Ascites and RE

Ascites was present in 672 patients with liver cirrhosis; 312 patients (46.4%) presented with RE. In the 207 patients without ascites, 66 (31.9%) had RE. There was a significant relationship between ascites and RE (χ2 = 5.76, P = 0.016).

#### EV size and RE

Of the patients with cirrhosis and EV, 44.7% (356/796) had RE. Twenty-two of 83 patients (26.5%) without EV had RE. Sixty-one of 158 patients (38.6%) with small size EV had RE. RE was found in 43.0% (163/379) and 51.0% (132/259) of patients with medium and large EV, respectively. There was no significant relationship between EV size and RE (χ2 = 2.6, P = 0.11).

## Discussion

GERD, which is divided into RE and Barrett's Esophagus, is common in Western countries [2,14]. The incidence of GERD is 5.77% in the general Chinese population [1]. It has been reported that the incidence of GERD is 65% in patients with decompensated cirrhosis [15]. In this study, the prevalence of RE, as confirmed by endoscopy, was 36.4% in patients with chronic liver disease, which was significantly higher than patients with acute hepatitis (10.3%). The prevalence of RE was 43.0% in patients with liver cirrhosis and 23.3% in patients with chronic hepatitis patients. The highest prevalence of RE existed between patients with liver failure or Child B and C liver cirrhosis. We also found that in patients with chronic liver disease, RE was more common in males (43.1%) than in females (21.4%). RE is becoming increasingly more prevalent in older patients with chronic liver disease. The prevalence of RE accounted for 63.1% in patients > 60 years of age; however, RE occurred in 20.0% of patients < 20 years of age with chronic liver disease. Therefore, there is a relationship between the high prevalence of RE among patients with chronic liver disease and severity of liver damage and age.

GERD clinical symptoms can be typical, such as dyspepsia and/or pyrosis. The typical symptoms of RE were present in 21.3% of those patients and mainly occurred in patients with chronic hepatitis (43%). However, most cirrhotic patients with RE were asymptomatic. Dyspepsia and pyrosis only accounted for 12.3% and 10.4%, of cirrhotic patients, respectively. Therefore, in chronic liver disease patients with RE, the clinician may not have mentioned this because of asymptomatic RE. Questioning patients about atypical reflux symptoms must be a part of the cirrhotic patient's history. If present, a work-up for GERD must be done with endoscopy or the patient should be empirically treated.

The causes and the mechanism of liver disease in patients with RE have not been elucidated. It has been confirmed that the high incidence of RE in patients with chronic liver disease is related to the following factors: ① There are changes in gastrointestinal hormones in patients with liver cirrhosis: Studies have shown that plasma vasoactive peptide and neurotensin in patients with liver cirrhosis are significantly higher than in the normal population, and vasoactive peptides and neurotensin are known to reduce the pressure of the lower esophageal sphincter (LES), which facilitates reflux of the stomach contents[5,7]. ② Emptying of the stomach is delayed: It has been reported that gastric half-emptying of liquid food is delayed in patients with liver cirrhosis, and the function of gastric emptying is influenced by the extent of liver function damage[16,17]. ③ Ascites induces an increase in intra-abdominal pressure, compressing the stomach and the stomach contents reflux[9,15]. ④ The EV influence esophageal emptying, so that the contact time of refluxed objects with the esophageal mucosa is extended[18]. ⑤ EV lead to LES dysfunction, which make the stomach contents reflux easily. It has recently been shown that the nitric oxide (NO) concentration is increased significantly in patients with liver disease, while NO is closely related to transient LES relaxation[19]. The current view showed that NO may be an activated factor of transient LES relaxation, which mediates esophageal LES smooth muscle relaxation and plays an important role in GERD. In this study, there was no relationship between ascites and RE.

GERD in cirrhotic patients with EV has been studied for many years, and great importance has been paid to esophageal dyspepsia as a risk factor for the rupture and bleeding of EV[18]. Other studies have reinforced these initial findings as they showed a higher prevalence of esophagitis and dyspepsia in cirrhotic patients with non-bleeding varices and a lower LES pressure in cirrhotic patients with massive ascites [9]. However, later studies have not supported this hypothesis because a lower LES pressure or a higher incidence of GERD was not demonstrated in this group of patients [15]. In the last few decades, as variceal bleeding continued to be a severe complication, new risk factors for rupture have been evaluated [19,20]. GERD was again studied and the prevalence among cirrhotic patients with EV has been studied [9,10]. Therefore, in cirrhotic patients, increased contact time between dyspepsia and EV could lead to the eventual erosion of the mucosa and EV bleeding. In our study, 44.7% of cirrhotic patients with varices had RE. The tendency of a high prevalence (51.0%) of RE was observed in large EV in cirrhotic patients (43.0% in medium and 38.6% in small EV). There was no significant relationship between EV size and RE.

## Conclusions

The high prevalence of RE detected during upper endoscopies in patients with chronic liver diseases has been demonstrated. There was no significant correlation between ascites, EV size, and high prevalence of RE in cirrhotic patients. It should be mentioned, however, that asymptomatic RE is more common in cirrhotic and liver failure patients. The role of RE in variceal bleeding, however, needs to be demonstrated.

## Competing interests

The authors declare that they have no competing interests.

## Authors' contributions

BZ and BL participated in clincial care, endoscopy, and drafted the manuscript. JWM and PL carried out endoscopy and clinical care. LL performed the data collection and statistical analysis. YMS participated in endoscopic assistance and nursing. HGD conceived the study, and participated in its design, coordination, and preparation of the final manuscript. All authors read and approved the final manuscript.

## Pre-publication history

The pre-publication history for this paper can be accessed here:

http://www.biomedcentral.com/1471-230X/10/54/prepub
